# Optimization of Decellularization Procedure in Rat Esophagus for Possible Development of a Tissue Engineered Construct

**DOI:** 10.3390/bioengineering6010003

**Published:** 2018-12-24

**Authors:** Panagiotis Mallis, Panagiota Chachlaki, Michalis Katsimpoulas, Catherine Stavropoulos-Giokas, Efstathios Michalopoulos

**Affiliations:** 1 Hellenic Cord Blood Bank, Biomedical Research Foundation Academy of Athens, 4 Soranou Ephessiou Street, 115 27 Athens, Greece; pmallis@bioacademy.gr (P.M.); giotachachlaki@gmail.com (P.C.); cstavrop@bioacademy.gr (C.S.-G.); 2 Center of Experimental Surgery, Biomedical Research Foundation Academy of Athens, 4 Soranou Ephessiou Street, 115 27 Athens, Greece; mkatsiboulas@bioacademy.gr

**Keywords:** esophagus, Barret’s esophagus, decellularization, CHAPS, SDS, histological images, tissue engineered construct

## Abstract

**Background:** Current esophageal treatment is associated with significant morbidity. The gold standard therapeutic strategies are stomach interposition or autografts derived from the jejunum and colon. However, severe adverse reactions, such as esophageal leakage, stenosis and infection, accompany the above treatments, which, most times, are life threating. The aim of this study was the optimization of a decellularization protocol in order to develop a proper esophageal tissue engineered construct. **Methods:** Rat esophagi were obtained from animals and were decellularized. The decellularization process involved the use of 3-[(3-cholamidopropyl) dimethylammonio]-1-propanesulfonate (CHAPS) and sodium dodecyl sulfate (SDS) buffers for 6 h each, followed by incubation in a serum medium. The whole process involved two decellularization cycles. Then, a histological analysis was performed. In addition, the amounts of collagen, sulphated glycosaminoglycans and DNA content were quantified. **Results:** The histological analysis revealed that only the first decellularization cycle was enough to produce a cellular and nuclei free esophageal scaffold with a proper extracellular matrix orientation. These results were further confirmed by biochemical quantification. **Conclusions:** Based on the above results, the current decellularization protocol can be applied successfully in order to produce an esophageal tissue engineered construct.

## 1. Introduction

Esophageal disease-related morbidity has increased dramatically in the last 10 years. More than 10,000 people have been affected by various types of esophageal disorder, such as congenital or acquired esophageal diseases, esophageal atresia and esophageal trauma [1,2]. Furthermore, over 500,000 individuals are diagnosed with esophageal cancer each year, worldwide [3,4]. For early stage esophageal malignancies and Barret’s esophagus, endoscopic mucosal resection (EMR) is the gold standard treatment [3]. However, most pathologies need segmental substitution of the esophagus with either autologous or synthetic grafts [5]. Autologous grafts from the stomach, jejunum or colon can be applied, but 40% of patients die due to serious adverse reactions, such as limited nutrition and esophagus infection [6].

Under this scope, a proper esophageal scaffold can be fabricated using the tissue engineering methods. Until now, many attempts have been performed in order to develop esophageal constructs utilizing polymer and synthetic materials, such as Dacron and expanded polytetrafluorethylene (ePTFE) [5,7,8]. Unfortunately, these constructs are accompanied by severe complications, such as anastomic leakage and esophageal stenosis. The esophagus is an organ that is located behind the trachea and consists of the epithelium, mucosa, submucosa and muscularis propria. Reproduction of this complicated structure with polymers or synthetic materials or even with 3D printing is extremely difficult and this may be the primary reason for construct failure [5,7,8]. On the other hand, the use of an acellular esophageal scaffold may be more effective than the aforementioned attempts [5]. Indeed, decellularized matrices have been used successfully in the experimental and clinical setting in the past for tissue replacement of a wide variety of organs such as the trachea, bladder, arteries and veins. Decellularization aims to remove tissue resident cells, while preserving the extracellular matrix (ECM) of the organ, reducing, in this way, the immunogenicity of the produced material. In addition, decellularized matrices are characterized by having greater biocompatibility rather than the artificial scaffolds [9]. The future goal is the production of an *off the shelf* esophageal scaffold, which can properly be implanted to human patients. 

Due to the complicated esophageal structure, the decellularization procedure must be established properly before the performance of any clinical attempts. Until now, a great effort has been performed by several groups worldwide in order to validate the decellularization procedure, but most of these studies have been accompanied by contradictory results regarding the preservation of ECM components in decellularized matrices [5,9,10,11,12]. In most of the above techniques, a combination of detergent and enzymatic treatments has been applied for successful cell removal. However, the esophagus is characterized by a collagen-rich ECM, which can be damaged by enzymes in an irreversible way. It is widely known that enzymes such as trypsin can cleave the collagen and elastin fibers, thus inducing severe damage to the tissue ECM. In addition, the extended use of anionic detergents such as sodium dodecyl sulfate (SDS) can damage the sulfated glycosaminoglycans (sGAGs), which are key components of the ECM. When crucial ECM components, such as collagen, elastin and sGAGs, are damaged, then the occurred decellularized constructs are characterized by totally different properties from the original ones; thus, their use as scaffold could be hampered [5,9,10,11,12,13].

The aim of this study was the validation of a previously described non-enzymatic decellularization protocol in rat esophagi (rES) [14,15]. For this purpose, decellularization of rES was evaluated after two cycles. Then, histological analysis, morphometric measurements and biochemical quantifications were performed. The future goal is the use of this protocol on esophagi derived from larger animals or cadaveric human donors to produce a proper esophageal tissue engineered construct that could be applied to the patients.

## 2. Materials and Methods

### 2.1. Preparation of Rat Esophagi

Esophagi were harvested under aseptic conditions from Sprague–Dawley rats (n = 30), weighing 250–300 g. All animals were provided by Biomedical Research Foundation Academy of Athens (BRFAA) and handled according to the guidelines of animal care which conform with the Helsinki declaration. In addition, this study was approved by the Bioethics Committee of BRFAA. Each harvested esophagus was rinsed in Phosphate Buffer Saline 1X (PBS 1X, Sigma-Aldrich, Darmstadt, Germany) and processed immediately to the decellularization procedure.

### 2.2. Decellularization of Rat Esophagi

rES (n = 10, l = 4 ± 1 cm) were cut into 3 segments of 1 cm and submitted to decellularization buffers. The decellularization process was performed according to previous described protocols with some modifications [12,13]. Briefly the esophagus segments were subjected to CHAPS buffer at pH 7 (8 mM CHAPS, 1 M NaCl and 25 mM EDTA in PBS 1X, Sigma-Aldrich, Darmstadt, Germany) for 6 h at room temperature under constant agitation at 350 rpm. Then, the esophageal segments were subjected to SDS buffer at pH 7, (1.8 mM SDS, 1M NaCl and 25 mM EDTA in PBS 1X, Sigma-Aldrich, Darmstadt, Germany) for additional 6 h at room temperature under constant agitation at 350 rpm. Finally, the esophageal segments were incubated in α-Μinimum Essentials Medium (α-ΜΕM, Sigma-Aldrich, Darmstadt, Germany) supplemented with 40% *v*/*v* Fetal Bovine Serum (FBS, Sigma-Aldrich, Darmstadt, Germany) for 6 h at 37 °C under constant agitation at 350 rpm. The above procedure was repeated for 1 more cycle. 

### 2.3. Histological Analysis

Native non decellularized (n = 10, l = 1 cm) and decellularized rES segments after the 1st (n = 10, l ≈ 1 cm) and 2nd (n = 10, l ≈ 1 cm) cycles were fixed in 4% *v*/*v* paraformaldehyde (PFA, Sigma-Aldrich, Darmstadt, Germany) for 4 h. Then, the samples were rehydrated, paraffin embedded and sectioned at 5 μm. The following histological stainings were performed in order to validate the effect of each decellularization cycle to the esophageal extracellular matrix. Hematoxylin and Eosin (H&E, Sigma-Aldrich, Darmstadt, Germany), Sirius Red (SR, Sigma-Aldrich, Darmstadt, Germany), Orcein Stain (OS, Sigma-Aldrich, Darmstadt, Germany) and Toluidine Blue (TB, Sigma-Aldrich, Darmstadt, Germany) were performed for the evaluation of the presence of cell nuclei, collagens, elastin and sGAGs, respectively. Images were acquired with a Leica DM L2 light microscope (Leica Microsystems, Weltzar Germany) and processed with Image J 1.46 (Wane Rasband, National Institute of Health, Bethesda, MD, USA).

Indirect immunofluorescence against fibronectin in combination with DAPI was performed in native and decellularized esophageal segments. Briefly, the slides were deparaffinized, rehydrated and blocked. Then, monoclonal antibody against rat fibronectin (1:5000, Sigma-Aldrich, Darmstadt, Germany) was added, incubated and followed by the addition of secondary FITC- conjugated mouse IgG antibody (1:100, Sigma-Aldrich, Darmstadt, Germany). Finally, DAPI was added in slides and incubated. The slides were mounted with glycerol and observed under a Leica SP5 II fluorescence microscope equipped with LAS Suite v2 software (Leica Microsystems, Weltzar, Germany). 

### 2.4. Morphometric Analysis

The morphometric analysis involved the measurement of length, mucosa thickness and total thickness in native and decellularized rES. Specifically, the length and total thickness were measured in native (n = 10) and decellularized rES after the 1st (n = 10) and 2nd (n = 10) cycles. Mucosa thickness was determined only in native (n = 10) and decellularized (n = 10) rES after 1st cycle. After the 2nd decellularization cycle, the rES ECM was damaged and thus, it could not be efficiently used to estimate mucosa thickness. The length of rES was determined with a digital caliper (Flip-Plus Electronic Caliper, Fowler, Newton, MA, USA). Mucosa thickness and total thickness measurements were performed in histological images, using Image J 1.46 (Wane Rasband, National Institute of Health, Bethesda, MD, USA).

### 2.5. Quantification of Collagen, sGAGs and DNA Content

Native (n = 10, l = 1 cm) and decellularized esophagi segments after the 1st (n = 10, l ≈ 1 cm) and 2nd (n = 10, l ≈ 1 cm) cycles were digested using a lysis buffer contained 0.1 M Tris pH 8, 0.2 M NaCl and 5 mM EDTA in PBS 1X (Sigma-Aldrich, Darmstadt, Germany) supplemented with 30 mg/mL Proteinase K (Sigma-Aldrich, Darmstadt, Germany). The digestion was performed at 56 °C for 12 h followed by inactivation at 90 °C for 5 min. 

The amount of collagen in each sample was quantified with a Hydroxyproline Assay kit (MAK008, Sigma-Aldrich, Darmstadt, Germany) according to the manufacturer’s instructions. SGAGs were measured by the addition of 1% *w*/*v* dimethylene blue (Sigma-Aldrich, Darmstadt, Germany), and photometric measurement was done at 525 nm. The concentration of sGAGs in each sample was obtained through interpolation to a standard curve. A standard curve was developed based on the chondroitin sulfate standards of 12 μg/mL, 25 μg/mL, 50 μg/mL, 100 μg/mL and 150 μg/mL. 

Finally, for the DNA quantification assay, the total genetic material from each sample was eluted in 100 μL of DNAse free water (Sigma-Aldrich, Darmstadt, Germany) followed by spectrophotometric quantification at 260 to 280 nm.

### 2.6. Statistical Analysis

Graph Pad Prism v 6.01 (GraphPaD Software, San Diego, CA, USA) was used for the statistical analysis. All parameters of this study were compared, using the Mann–Whitney test. Statistically significant differences between group values were considered when the *p*-value was less than 0.05. Indicated values are presented as mean ± standard deviation.

## 3. Results

### 3.1. Histological Analysis

**Figure 1:**
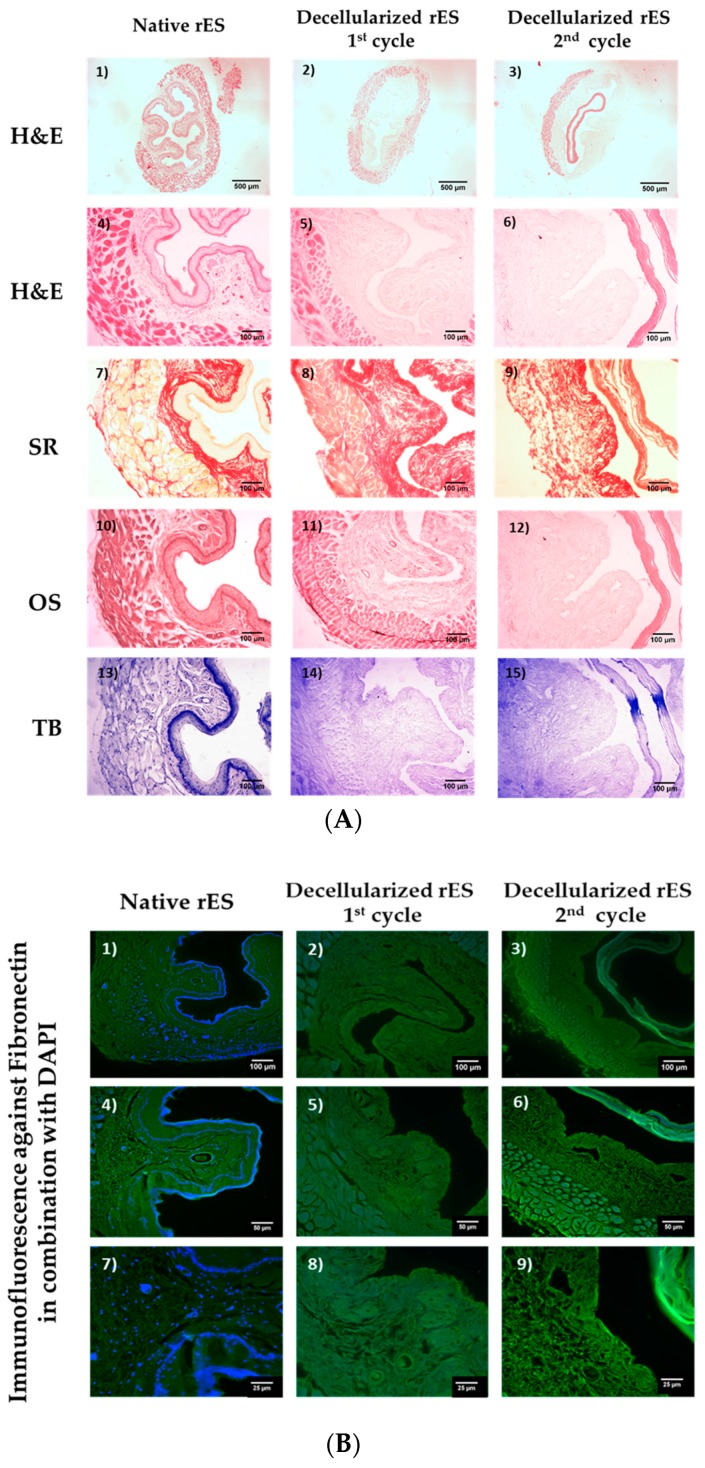
Histological analysis of decellularized rat esophagi (rES) after the first and second cycles. Native rES stained with H&E (**A1**,**A4**), SR (**A7**), OS (**A10**) and TB (**A13**). Decellularized rES stained with H&E (**A2**,**A3**,**A5**,**A6**), SR (**A8**,**A9**), OS (**A11**,**A12**) and TB (**A14**,**A15**) after the first and second cycles, respectively. The black arrows indicate elastin preservation in decellularized esophagi after the first cycle. Images A1–A3 are presented with original magnification 2.5×; scale bars are 500 μm. Images A4–A15 are presented with original magnification 10×; scale bars are 100 μm. Indirect immunofluorescence against fibronectin in combination with DAPI in native (**B1**,**B4**,**B7**) and decellularized rES after the first (**B2**,**B5**,**B8**) and second (**B3**,**B6**,**B9**) cycles. Images B1–B3 are presented with original magnification 10×; scale bars are 100 μm. Images B4, B5 and B6 are presented with original magnification 20×; scale bars are 50 μm. Images B7, B8 and B9 are presented with original magnification 40×; scale bars are 25 μm.

rES were successfully decellularized with the current protocol. After the first decellularization cycle, rES were characterized by well-preserved ECM, while cellular and nuclear materials were eliminated (Figure 1). Decellularized rES after the second cycle presented extensive damage in their ECMs. 

Specifically, decellularized rES after the first cycle successfully retained their matrix components, such as collagen, elastin and sGAGs, as indicated by SR, OS and TB stains (Figure 1). Indeed, the SR stain revealed the preservation of the collagen fibers in decellularized rES after the first cycle. Moreover, decellularized rES appeared to have a more compact structure when compared to native samples. This phenomenon might be a result of cell loss during the decellularization procedure. Elastin fibers were stained black by OS, thus revealing their intact structure in decellularized rES after the first cycle. Finally, decellularized rES after the first cycle were characterized by a weaker TB stain as compared with the native rES.

On the other hand, decellularized rES after the second cycle, presented wide destruction of ECM key components. Indeed, the inner layer of decellularized rES after the second cycle was totally detached from the rest of the esophageal matrix (Figure 1). In addition, collagen fibers appeared to be damaged in decellularized rES after the second cycle, as was indicated by the weaker SR stain when compared to native samples. Elastin was absent in decellularized esophagi after the second cycle, whereas sGAGs did not present any significant alteration. 

Indirect immunofluorescence results showed the preservation of well oriented fibronectin in rES after the first cycle (Figure 1 and Figure S1). RES from the second decellularization cycle were characterized by damaged fibronectin (Figure 1). DAPI stain was observed only in native samples. No DAPI stain was evident in decellularized rES either from the first or second cycle (Figure 1). The indirect immunofluorescence results regarding the preservation or damage of ECM components appeared to be consistent with the histological staining results. The above results strongly indicate that one cycle is enough to produce a proper decellularized rES without further damage to its ultrastructure.

### 3.2. Morphometric Analysis

Further validation of the current decellularization protocol to rES involved a morphometric analysis. For this purpose, histological images were used in order to measure the mucosa thickness and the total thickness, while the total esophageal length was measured with a digital caliper. 

**Figure 2:**
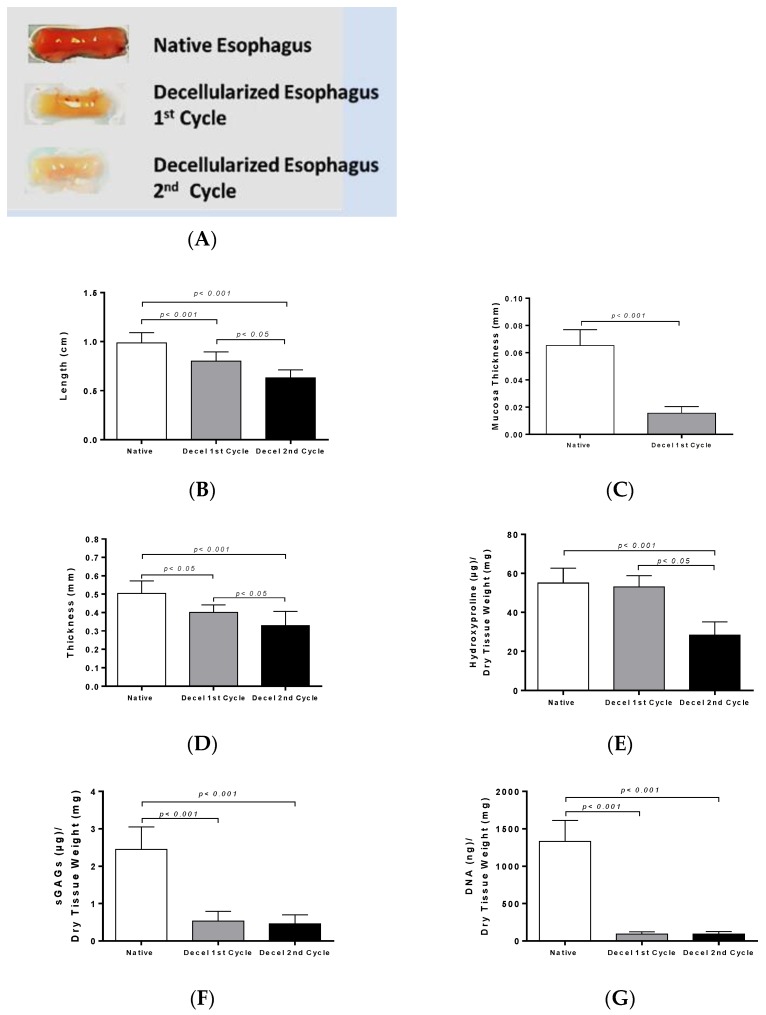
Morphometric analysis and biochemical quantification in rES. Macroscopic overview of native and decellularized rES after the first and second cycles (**A**). Measurement of length (**B**), mucosa thickness (**C**) and total thickness (**D**) in rES. Statistically significant differences were observed in the above parameters between native and decellularized rES either by the first (*p* < 0.005) or second cycle (*p* < 0.001); Biochemical quantification involved hydroxyproline measurement (**E**); sulfated glycosaminoglycans (sGAGs) (**F**) and DNA (**G**) content determination. Statistically significant differences were observed in collagen, sGAG and DNA content between native and decellularized rES either by the first (*p* < 0.05) or second cycle (*p* < 0.05).

The length of decellularized rES after the first cycle reduced by 19% and after the second cycle by 36%. Specifically, the length of native non-decellularized rES was 1.0 ± 0.1 cm, while the length of decellularized rES after the first and second cycles was 0.8 ± 0.1 cm and 0.6 ± 0.1 cm, respectively (Figure 2). This decrease in length between native and decellularized esophagi from both cycles was found to be statistically significant (*p* < 0.001).

Native rES consists of epithelium, mucosa, submucosa and muscularis propria (Figure S2). Among them, mucosa thickness and total thickness were measured.

Specifically, the total thickness was 0.5 ± 0.1 mm in native rES, 0.4 ± 0.1 mm in decellularized rES after the first cycle and 0.3 ± 0.1 cm after the second cycle (Figure 2). Statistically significant differences were observed in total thickness between the native and decellularized rES after the first (*p* < 0.05) and second cycles (*p* < 0.001). Furthermore, statistically significant differences were observed in the total thickness between decellularized rES of the first and second cycles (*p* < 0.05). Finally, the thickness of mucosa layer in native and decellularized rES was measured. The thickness of mucosa layer was 65 ± 1 μm in native rES and 15 ± 1 μm in decellularized rES after the first cycle, while this layer totally damaged after the second decellularization cycle and could not be measured (Figure 2).

### 3.3. Biochemical Analysis and DNA Quantification

Collagen, sGAG and DNA content were quantified in order to validate the current decellularization protocol. Specifically, native rES characterized by 55.1 ± 7.3 μg hydroxyproline per mg of dry tissue weight, while decellularized rES after the first and second cycles were characterized by 53.2 ± 5.5 and 28.1 ± 6.4 μg hydroxyproline per mg of dry tissue weight, respectively (Figure 2). No statistically significant difference was observed in the collagen amount between native and decellularized rES after the first cycle. Statistically significant differences were observed between native and decellularized rES after the second cycle (*p* < 0.001) and decellularized rES from the first to the second cycle (*p* < 0.05). 

SGAG content was significantly reduced between native and decellularized rES from both cycles. The SGAG content in native rES was 2.4 ± 0.5 μg sGAG per mg tissue weight. Decellularized rES after the first and second cycles were characterized by 0.5 ± 0.2 and 0.4 ± 2 μg sGAG per mg of dry tissue weight, respectively (Figure 2).

The DNA amount in native rES was 1300 ± 268 ng DNA per μg of dry tissue weight, while after the first decellularization cycle, it was 93 ± 26 ng DNA per μg of dry tissue weight and after the second decellularization cycle, it was 93 ± 29 ng DNA per μg of dry tissue weight (Figure 2). Statistically significant differences were observed between native and decellularized rES either by the first (*p* < 0.001) or second cycle (*p* < 0.001).

## 4. Discussion

Esophagus-related diseases and their postoperatively complications are associated with a high mortality rate [1,2,3,4,5]. These diseases affect either pediatric or adult patients and, most times, the use of autografts derived either from jejunum or gastric interposition or synthetic grafts are the gold standard treatments [3,4,5]. Unfortunately, severe complications are frequently observed and can be life threating for the patients. 

Esophagus tissue engineering is a promising solution, although it is still challenging, and more effort must be performed in this direction. The aim of this study was to validate a decellularization protocol that has been previously used successfully in other tissues, such as human umbilical arteries and porcine pericardium [14,15,16]. The goal of this study was to produce a proper esophageal scaffold, reducing the ECM damage and the processing time. For this purpose, histological analysis, morphometric measurements and quantification of collagen, sGAGs and DNA were performed.

The histological analysis revealed the successful decellularization of rES using only one decellularization cycle. Indeed, after the first decellularization cycle, rES were characterized by a properly organized ECM, collagen, elastin and sGAGs were retained, while total cellular and nuclear materials were lacking. However, after the second decellularization cycle, rES were extensively damaged. The inner mucosa layer appeared to be detached from the rest of the esophageal matrix. Moreover, these results are similar to previous reports from other groups, thus indicating the success of the current decellularization protocol [9,10,11,17,18]. In most of these studies, an increased number of decellularization cycles or increased processing time was required. In our study, only one decellularization cycle was needed to successfully decellurize the rES. Specifically, in the study of Urbani et al. [18], three decellularization cycles were used in order to completely remove the cellular and nuclear materials. In Urbani’s study, even after the first decellularization cycle, the esophagus was characterized by damaged collagen and elastin fibers. Unlike these results, in our experimental procedure, the histological analysis indicated well-organized esophageal ECMs with no cellular and nuclear materials and without the need for a second decellularization cycle. This discrepancy in the results between these two studies might be attributed to the different origins of the esophagi and decellularization protocol that were used. 

The histological analysis also involved indirect immunofluorescence against fibronectin in combination with DAPI staining in native and decellularized rES. Fibronectin appeared to be well preserved in rES after the first decellularization cycle, while the rES of the second cycle were characterized by damaged fibronectin. No DAPI stain was evident in decellularized rES, thus further confirming our initial histological results regarding the absence of nuclear materials. These results are in accordance with the study by Bhrany et al., indicating the successful preservation of fibronectin after decellularization [9]. Fibronectin plays a significant role in the epithelium maintenance and function through its binding sites. The preservation of fibronectin in decellularized rES is of major importance, as it makes them efficient for re-epithelization.

The morphometric analysis revealed that the length, mucosa thickness and total thickness of the rES were significantly decreased after each decellularization cycle. As a consequence of these morphometric changes, biomechanical alterations may be revealed, as has been reported in previous studies [9,11]. In a number of studies including decellularized matrices from various origins, such as vessels and aortic valves, the thickness was increased [10,17,18]. In most of these studies, thickness measurements were performed with caliper instruments in non-formalin fixed decellularized matrices. It is known that decellularized matrices attract water molecules, thus increasing their total weights and enlarging their wall thicknesses. In order to validate the thickness change between native and decellularized tissues, the initial water content of native tissues must be determined. Any attempt to perform the above measurements in non-fixed native and decellularized matrices will be not be accurate enough. In order to avoid this phenomenon, in our study, mucosa and total thickness were measured from histological images using image analysis software. In this way, the same dehydration rate was achieved both in native and decellularized tissues, obtaining, in this way, more accurate results than the aforementioned studies [10,18,19].

The next step of this study was biochemical quantification which involved collagen, sGAGs and DNA determination. The collagen content did not present any statistically significant changes between native and decellularized rES after the first and second cycles. On the contrary, sGAGs were decreased after the first decellularization cycle, followed by an extensive decrease after the second decellularization cycle. This decrease in sGAG content between native and decellularized rES (first and second cycles) was statistically significant. SGAGs form large macromolecules called proteoglycans and are responsible for the collagen orientation in tissues. SDS, a reagent that was used in the decellularization protocol, can harm the sGAGs through binding to their negatively-charged sites. This decrease in sGAG content might alter the collagen orientation, thus damaging the tissue ECM. However, no structural alterations of ECM in decellularized rES after the first cycle were observed as indicated by the histological analysis. The wide damage that was observed in the ECM of rES after the second cycle, was possibly due to the greater impact of the decellularization reagents to all structural tissue components (collagen, elastin, fibronectin and sGAGs) and not specifically to sGAGs. 

Further confirmation of the successful removal of genetic material from decellularized rES was performed by DNA quantification. DNA content was reduced by over 95% in decellularized rES from both cycles. The above results are comparable with previous works in other tissues and further confirm that one decellularization cycle is more than enough to produce an esophageal tissue engineered construct. 

Under this scope, decellularized rES was characterized by a properly preserved ECM with no cellular or nuclear material, as indicated by the histological stains. Moreover, histological and biochemical analysis results were found to be consistent with the criteria of successful tissue decellularization that were provided by the study of Crapo et al. [13,20]. In this way, and based on the above results, strong evidence is provided regarding the successful decellularization of rat esophagi with the proposed decellularization protocol [13,20].

## 5. Conclusions

The aim of this study was to optimize a decellularization protocol for proper development of an esophageal tissue engineered construct. Unlike previous published studies [9,10,11,15,16], our decellularization protocol is cost effective and less time consuming, producing a decellularized matrix with the same structure and function properties. In order to further confirm the proper preservation of ECM components and their properties as an esophageal scaffold, more experiments need to be performed, including a cytotoxicity assay, biomechanical testing, recellularization with tissue specific cell populations (epithelial cells and muscle cells) and implantation to animal models. The future goal of this study is the use the of current decellularization protocol in esophagi from larger animal models and from cadaveric human donors in order to develop a proper esophageal tissue engineered construct. This construct could be applied in patients, eliminating the use of autologous stomach, intestine conduits or synthetic grafts, thus lowering the morbidity which is caused by the adverse side effects of the above applications.

## Supplementary Materials

The following are available online at http://www.mdpi.com/2306-5354/6/1/3/s1, Figure S1: Indirect immunofluorescence against fibronectin in combination with DAPI stain in native and decellularized rES. Figure S2: Histological image of native and decellularized rat esophagus with H&E.
