# Supplementary material for: Optimization of Decellularization Procedure in Rat Esophagus for Possible Development of a Tissue Engineered Construct
Source: Bioengineering (Basel). 2018 Dec 24;6(1):3. doi: 10.3390/bioengineering6010003 (PMC6466343; doi:10.3390/bioengineering6010003)
Supplement: Supplementary file 1 [file bioengineering-06-00003-s001.pdf]

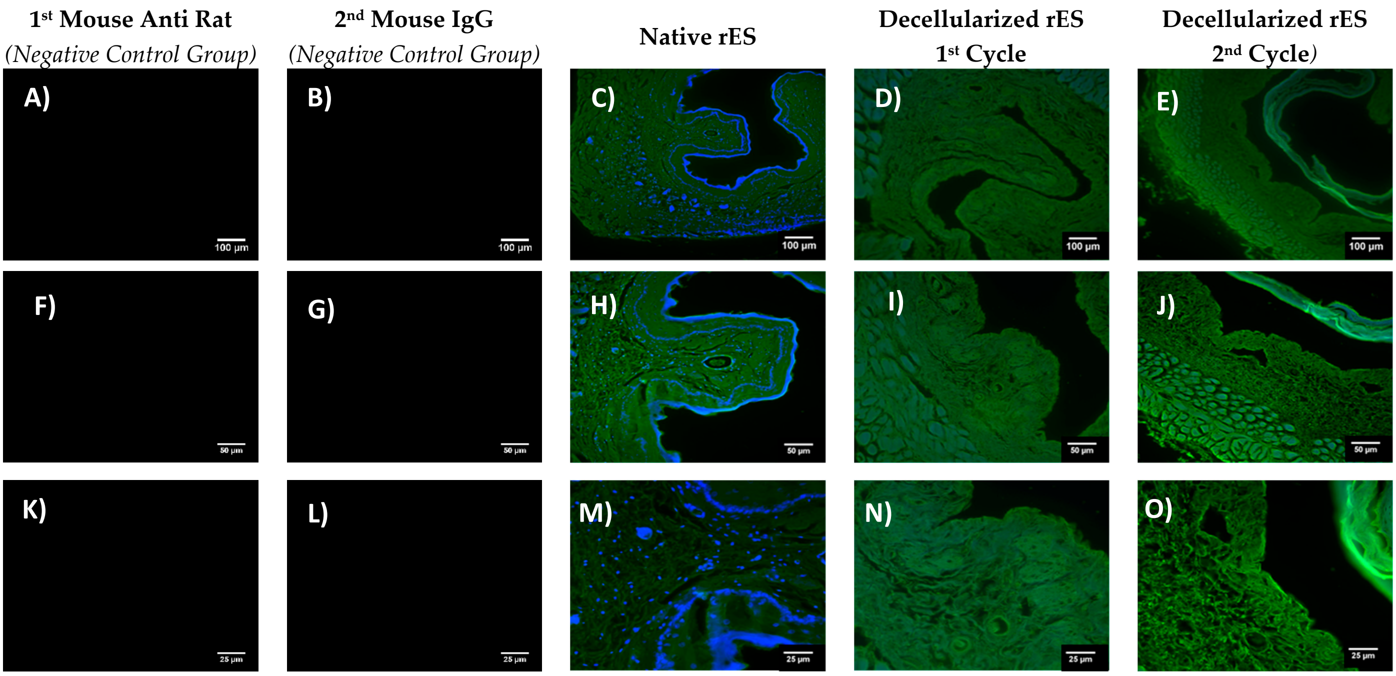

Figure S1. Indirect immunofluorescence against fibronectin in combination with DAPI stain in native and decellularized rES. 1st Mouse Anti-Rat against fibronectin (negative control group, A,F and K). 2nd IgG (negative control group, B, G and L). Indirect immunofluorescence against fibronectin in combination with DAPI in native (C, H and M) and decellularized rES after 1st cycle (D, I and N) and after 2nd cycle (E, L and O). Images A-E, were acquired with original magnification 10x, scale bars 100 µm. Images F- J, were acquired with original magnification 20x, scale bars 50 µm. Images K- O, were acquired with original magnification 40x, scale bars 25 µm.

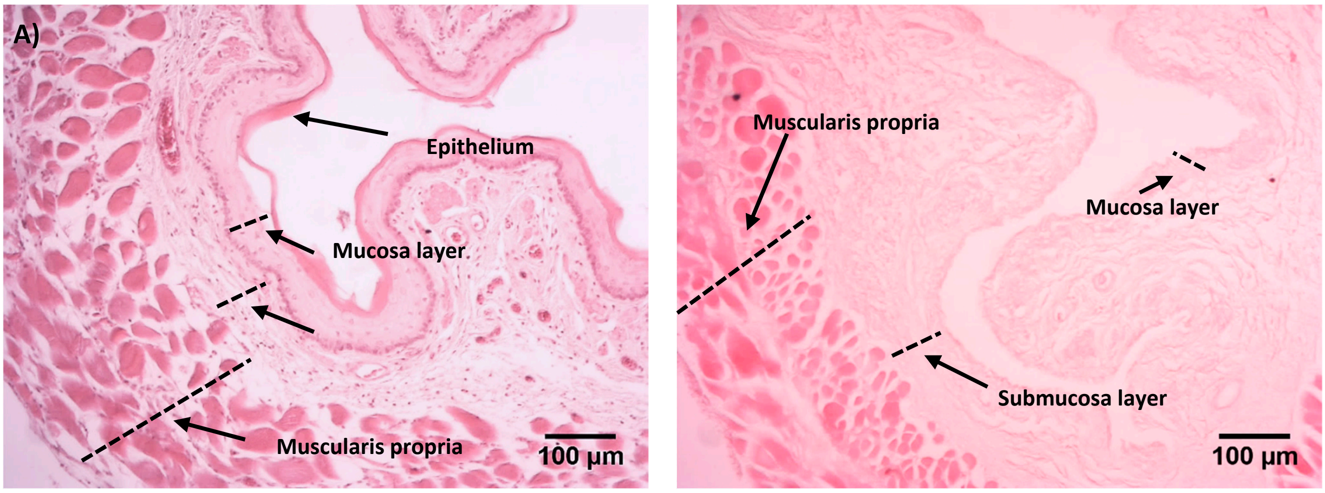

Figure S2. Histological image of native and decellularized rat esophagus with H&E. A) Native rat esophagus stained with H&E. B) Decellularized rat esophagus after 1st cycle stained with H&E. Rat esophagus is consisted of epithelium, mucosa, submucosa and muscularis propria which are visible in native sample. These layers and especially mucosa appeared to be decreased after the decellularization procedure. Original magnification 10x, scale bar 100 µm.
